# Increased frequency of integrons and β-lactamase-coding genes among extraintestinal *Escherichia coli* isolated with a 7-year interval

**DOI:** 10.1007/s10482-012-9797-9

**Published:** 2012-09-04

**Authors:** Joanna Mokracka, Anna Oszyńska, Adam Kaznowski

**Affiliations:** Faculty of Biology, Department of Microbiology, Adam Mickiewicz University in Poznań, ul. Umultowska 89, 61-614 Poznań, Poland

**Keywords:** Integrons, ESBL, Antibiotic resistance, ExPEC

## Abstract

We analyzed the level of antimicrobial resistance, and the presence of integrons and β-lactamase-coding genes in 69 clinically relevant *Escherichia coli* strains originating from extraintestinal infections isolated in 1999–2001 and 2008–2010. Comparison of the two groups showed significant differences in drug resistance frequency, and the presence of integron and β-lactamase-coding genes. The frequency of resistance to all antimicrobials beside imipenem, streptomycin, piperacillin/tazobactam, and sulfamethoxazole increased significantly, especially towards aminoglycosides, β-lactams and fluoroquinolones. Similarly, we noticed an increase in the number of strains with integrons from 31.6 to 80.7 %. The presence of integrase genes was associated with elevated frequency of resistance to each antimicrobial tested besides imipenem, piperacillin/tazobactam and ceftazidime. The presence of integrons was also associated with multidrug resistance phenotype. The genetic content of integrons comprised genes determining resistance toward aminoglycosides, sulfonamides and trimethoprim. Moreover, we noticed a significant increase in the frequency of *bla*_CTX-M_ β-lactamases, with appearance of *bla*_CTX-M-15_ variant and newer plasmid-encoded β-lactamases like CMY-15 and DHA. The emergence of strains resistant to several classes of antimicrobials and carrying integrons, ESBL and AmpC β-lactamase-coding genes may predict the spread of isolates with limited treatment options.

**Electronic supplementary material:**

The online version of this article (doi:10.1007/s10482-012-9797-9) contains supplementary material, which is available to authorized users.

## Introduction

*Escherichia coli* is a facultative Gram-negative species commonly present as a commensal organism in intestinal tract of mammals, but also recognized as one of major pathogens of human and animals. Pathogenic *E. coli* strains capable of causing disease outside the intestinal tract are classified as extraintestinal pathogenic strains (ExPEC) (Russo and Johnson 2000; Kaper et al. 2004). ExPEC are divided into isolates causing urinary tract and bloodstream infections (uropathogenic strains, UPEC), and neonatal septicemia/meningitis (MNEC, meningitis associated *E. coli*) strains (Welch 2006). During the past years, the rates of antimicrobial resistance among ExPEC strains have increased substantially, leading to higher morbidity and mortality and substantially increasing treatment costs (Daikos et al. 2007). Cephalosporins, fluoroquinolones, and co-trimoxazole are often used to treat ExPEC infections and the surveillance studies in years 2000–2010 indicated that 20–40 % strains become resistant to that group of antimicrobials (Pitout 2012).

Antibiotic resistance may develop through mutations in chromosomal DNA or acquisition of plasmids or transposons carrying resistance determinants. Integrons play an important role in the spread of antimicrobial resistance of clinical *Enterobacteriaceae* strains, since they capture, integrate and express gene cassettes encoding proteins associated with antimicrobial resistance. The integron covers DNA fragment that consists of an integrase gene of the tyrosine recombinase family, primary recombination site called the *attI*, and a promoter P_C_ that directs transcription of the captured genes (Hall and Collis 1995; Mazel 2006). Integrons are often associated with mobile DNA elements like transposons and plasmids, which enable lateral spread of resistance determinants. Five classes of integrons are recognized on the basis of integrase gene sequence (Cambray et al. 2010). All classes are associated with resistance determinants, three of them are responsible for multidrug resistance (MDR), with class 1 being most ubiquitous among clinical strains (Leverstein-van Hall et al. 2003; Mokracka et al. 2011). Over 130 different gene cassettes have been identified within integrons, providing resistance to most classes of antimicrobials, including β-lactams, aminoglycosides, amphenicols, macrolides, trimethoprim, quinolones and antiseptics (Partridge et al. 2009; Cambray et al. 2010). *Enterobacteriaceae* strains harbour numerous integron-embedded antimicrobial resistance determinants, including β-lactamase-coding genes (Weldhagen 2004; Eckert et al. 2006).

The aim of this research was to analyze the resistance patterns and characterize distribution and genetic content of integrons and β-lactamase encoding genes within the *E. coli* strains isolated from extraintestinal infections in the course of last decade.

## Materials and methods

### Clinical specimens

Sixty-nine clinically relevant strains of *E.* *coli* were isolated from specimens from inpatients of Poznań hospitals. Thirty-eight strains were collected in 1999–2001 (Group 1) and thirty-one from December 2008 to May 2010 (Group 2). The organisms were grown on MacConkey agar no. 3 (Oxoid) and identified with API 20E (bioMérieux) as *E. coli*. They were isolated from urine (32 strains), throat swab (10), vaginal swab (7), blood (5 strains), broncho-alveolar lavage (4), semen (3), eye ventricle (2), and single strains from cerebrospinal fluid, wound, sputum, tracheostomy tube, ulceration and abscess.

The strains were stored in −80 °C in BHI/glycerol (50/50). All phenotypic assays and determination of antimicrobial resistance were done immediately after collection. The interpretation of zone diameters was done according to the CLSI (2009) breakpoints.

### Clonal analysis by ERIC-PCR

The ERIC-PCR method utilizes primers complementary to enterobacterial repetitive intergenic consensus sequences of genomic DNA. The PCR reaction with primers ERIC 1 and ERIC 2 were done according to Versalovic et al. (1991). Computer analysis of electrophoretic patterns was carried out using GelCompar II version 3.5 software (Applied Maths). Similarity between fingerprints was calculated with the Dice coefficient. Cluster analysis was performed using the unweighted pair-group method with average linkages (UPGMA).

### Antimicrobial susceptibility

The susceptibility to 29 antibiotics representing 11 classes was determined according to the standard disc diffusion method recommended by Clinical and Laboratory Standards Institute guidelines (CLSI 2009). The antimicrobials included: amikacin (30 μg), tobramycin (10 μg), netilmicin (30 μg), gentamicin (10 μg), kanamycin (30 μg), streptomycin (10 μg), ampicillin (10 μg), ticarcillin (75 μg), ciprofloxacin (5 μg), norfloxacin (10 μg), tetracycline (30 μg), amoxicillin/clavulanic acid (30 μg), cefotaxime (30 μg), ceftazidime (10 μg), cefuroxime (10 μg), cefoperazone (10 μg), cefazolin (10 μg), cephalothin (30 μg), cefepime (30 μg), cefoxitin (30 μg), sulfamethoxazole (25 μg), co-trimoxazole (25 μg), trimethoprim (5 μg), piperacillin (100 μg), piperacillin/tazobactam (110 μg), chloramphenicol (30 μg), aztreonam (30 μg), nitrofurantoin (300 μg), and imipenem (10 μg). All strains exhibiting intermediate resistance zones were considered resistant. Production of extended spectrum β-lactamases (ESBL) was checked by the double-disc synergy test with ceftazidime, cefotaxime and amoxicillin/clavulanic acid.

### Identification of *bla* genes

Genes coding for extended spectrum β-lactamases and AmpC lactamases: *bla*_CTX-M_, *bla*_SHV_, *bla*_TEM_, *bla*_OXA_, *bla*_GES_, *bla*_VEB_, *bla*_PER_, *bla*_DHA_ and *bla*_CMY_ were identified by PCR method. The sequences of primers targeting β-lactamase genes were published elsewhere (Li CR et al. 2003; Li Y et al. 2008; Sáenz et al. 2004; Mendonça et al. 2008; De Champs et al. 2002; Aragón et al. 2008; Gniadkowski et al. 1998a, b). PCR amplifications were performed in a 25-μl volume with 2.5 μl of 10× PCR buffer with NH_4_(SO_4_)_2_, 0.6 μM of each primer, 100 μM of dNTP mix, 2.5 mM of MgCl_2_, 1 U of Hi-Fi *Taq* polymerase (Novazym), and 200 ng of genomic DNA. Amplification involved an initial denaturation (94 °C, 5 min) followed by 30 cycles of denaturation (94 °C, 30 s), annealing (55 °C, 30 s for *bla*_CTX-M_ and *bla*_SHV_, 50 °C 1 min for *bla*_TEM_, *bla*_OXA_, *bla*_GES_, *bla*_VEB_, *bla*_CMY_, 48 °C 1 min for *bla*_PER_, and 60 °C 30 s for *bla*_DHA_), and extension (72 °C, 1 min), with a final extension step (72 °C, 8 min). Variants of *bla*_TEM_, *bla*_CTX-M_, *bla*_OXA_, *bla*_SHV_, *bla*_CMY_, and *bla*_DHA_ were identified upon comparing the sequences with available GenBank sequence data by using ClustalW and the neighbor-joining method.

The genetic environment of *bla*_CTX-M_ was determined with primers sets targeting ISCR1 (formerly orf513), and IS*Ecp1* (Eckert et al. 2006; Quiroga et al. 2007).

### Analysis of integrons

Multiplex PCR was done for identification of integron integrase genes. PCR amplification was performed in a 25-μl volume with 2.5 μl of 10× PCR buffer with NH_4_(SO_4_)_2_, 0.25 μM of each primer, 100 μM of dNTP mix, 2.5 mM of MgCl_2_, 1 U of Hi-Fi *Taq* polymerase, and 200 ng of genomic DNA. Amplification involved an initial denaturation (94 °C, 5 min) followed by 30 cycles of denaturation (94 °C, 1 min), annealing (59 °C, 1 min) and extension (72 °C, 1 min), with a final extension step (72 °C, 8 min). The sequences of primers targeting *intI1*, *intI2* and *intI3* genes were recommended by Dillon et al. (2005).

Variable regions of class 1 and class 2 integrons were analyzed by conserved segment PCR (CS-PCR) and Hep-PCR, respectively. Sequences of primers complementary to the 5′ and 3′conserved regions of 1 class integrons (5′-CS and 3′-CS) and class 2 integron (Hep74 and Hep51) were published elsewhere (Lévesque et al. 1995; White et al. 2001). PCR amplifications were conducted as follow: initial denaturation 94 °C, 5 min, and 30 cycles of 94 °C 1 min, 55 °C 1 min, 72 °C 5 min, and final elongation 72 °C 8 min.

To check the gene cassette content, CS-PCR and Hep-PCR products were purified and sequenced. In case of two amplicons and amplicons longer than 1.7 kbp, the products were cloned by using pGEM^®^-T Easy Vector (Promega). Sequence data were analyzed with DNA Baser (HeracleSoftware) and aligned with available GenBank data using BLASTn. A gene cassette was identified if the percentage of similarity with GenBank data was higher than 95 %.

All PCR reactions were performed in a C1000 Thermal Cycler (BioRad). The PCR products were separated in 1.5 % agarose gel (Novazym). Molecular weight of PCR products was determined by Bio-Capt v. 99.04 software (Vilber Lourmat). All experiments were done in triplicate.

### Statistical analysis

Association between the frequency of antibiotic resistance and integron presence was calculated using Pearson’s χ^2^ test and Fisher’s exact test. Association between integron presence and resistance ranges was determined with the Mann–Whitney *U* test (Statistica 10, StatSoft). *P* < 0.05 was considered to indicate statistical significance.

## Results

### Clonal analysis by ERIC-PCR

The fingerprints of *E. coli* isolates consisted of 1–17 bands ranging in size from 110 to 5,000 bp (Supplementary Fig. S1). Three pair of isolates (EC1 and EC3, EC45 and EC53, EC49 and EC62) had ERIC-PCR profiles with 100 % similarity, yet analysis of integrons and resistance patterns indicated differences between them. Strains EC1 and EC3 had different resistance patterns, EC45 and EC53 differed in *bla* gene content, whereas EC49 and EC62 had also different integron gene cassette arrays (Table 1).

**Table 1:** Integrons, β-lactamase-coding genes and resistance patterns of *E. coli* isolates

Strain	Origin	Integrase	Integron variable region size (kbp)	Integron’s gene cassettes	*bla* genes	Resistance pattern
1999–2001 (38 isolates)						
EC1	Urine	–	–	–	–	KAN, STR, AMP, FOX, CEF, SUL, ATM
EC2	Blood	–	–	–	–	STR, CEF, SUL
EC3	Urine	–	–	–	–	KAN, STR, AMP, FEP, FOX, CEF, SUL, SXT, CHL
EC4	Blood	*intI1*	1.6	*dfrA1*-*aadA1*	*bla*_TEM-1_	AMK, GEN, STR, AMP, PIP, TIC, AMC, FEP, CFP, CXM, CEF, SUL, TMP, SXT, TET, CHL
EC5	Cerebrospinal fluid	–	–	–	–	GEN, STR, AMP, TZP, FEP, CEF, SUL, TET
EC6	Blood	*intI1*	1.7	*dfrA17*-*aadA*5	*bla*_TEM-1_	AMK, GEN, NET, TOB, STR, AMP, PIP, TIC, AMC, FEP, CFP, FOX, CXM, CEF, SUL, TMP, SXT, TET, CHL, NIT
EC7	Eye ventricle swab	–	–	–	–	NET, STR, AMP, TZP, TIC, FEP, CTX, CAZ, CEF, CIP, TMP, TET, NIT
EC8	Urine	–	–	–	*bla*_TEM-1_	STR, AMP, PIP, TIC, AMC, CFZ, CFP, CTX, FOX, CEF, SUL
EC9	Urine	*intI1*	1.9	*dfrA12*-*orfF*-*aadA2*	*bla*_CTX-M-3_	AMK, GEN, KAN, NET, STR, TOB, AMP, PIP, TIC, AMC, CFZ, FEP, CFP, CTX, CXM, CEF, SUL, TMP, SXT, TET, ATM
EC10	Vaginal swab	–	–	–	–	AMK, GEN, STR, FOX, SUL
EC11	Urine	–	–	–	–	STR, AMP, FEP, CFP, CEF, SUL, TMP
EC12	Blood	–	–	–	*bla*_TEM-1_	STR, AMP, PIP, TIC, AMC, CFZ, CFP, CTX, CEF, TET
EC13	Urine	–	–	–	–	STR, TET
EC14	Urine	–	–	–	–	STR, SUL
EC15	Urine	–	–	–	–	KAN, STR, SUL
EC16	Urine	–	–	–	–	STR, PIP, SUL, TET
EC17	Urine	–	–	–	–	AMK, TOB, CAZ, CEF, SUL
EC18	Semen	–	–	–	*bla*_TEM-1_	STR, AMP, TIC, SUL, TET
EC19	Urine	–	–	–	–	CEF, CIP, SUL
EC20	Semen	–	–	–	–	STR, CIP, NOR, SUL
EC21	Urine	–	–	–	–	STR, TOB, AMP, CEF, SUL, TMP
EC22	Urine	–	–	–	–	STR, CFZ, CIP, SUL
EC23	Semen	–	–	–	–	TZP, FEP
EC24	Urine	–	–	–	*bla*_TEM-1_	AMK, STR, TOB, CXM, SUL, TET
EC25	Urine	–	–	–	–	STR, SUL, TET
EC26	Eye ventricle swab	–	–	–	–	STR, SUL, TET
EC27	Brain abscess	–	–	–	*bla*_SHV-1_	AMP, PIP, TIC, CAZ, SUL
EC28	Throat swab	*intI1*	1.6	*dfrA1*-*aadA1*	–	KAN, STR, PIP, CFZ, SUL, TMP, SXT
EC29	Throat swab	–	–	–	–	KAN, STR, PIP, AMC, CEF, SUL, TMP, TET
EC30	Urine	*intI1*	1.6	*dfrA1*-*aadA1*	–	AMK, KAN, STR, CFP, SUL, TMP, SXT
EC31	Urine	*intI1*	1.9	*dfrA12*-*orfF*-*aadA2*	*bla*_CTX-M-3_	AMK, GEN, KAN, NET, STR, TOB, AMP, PIP, TIC, AMC, CFZ, FEP, CFP, CTX, CXM, CEF, SUL, TMP, SXT, TET, ATM
EC32	Throat swab	*intI1*	1.9	*dfrA12*-*orfF*-*aadA2*	*bla*_CTX-M-3_	AMK, GEN, KAN, NET, STR, TOB, AMP, PIP, TIC,, CFZ, FEP, CFP, CTX, CXM, CEF, SUL, TMP, SXT, TET
EC33	Urine	*intI1*	1.9	*dfrA12*-*orfF*-*aadA2*	*bla*_CTX-M-3_	AMK, GEN, KAN, NET, STR, TOB, AMP, PIP, TIC, CFZ, FEP, CFP, CTX, CXM, CEF, CIP, SUL, TMP, SXT, TET, ATM
EC34	Throat swab	*intI1*	1.9	*dfrA12*-*orfF*-*aadA2*	*bla*_CTX-M-3_	AMK, GEN, KAN, NET, STR, TOB, AMP, PIP, TIC, CFZ, FEP, CFP, CTX, CXM, CEF, SUL, TMP, SXT, TET, ATM
EC35	Throat swab	*intI1*	1.9	*dfrA12*-*orfF*-*aadA2*	*bla*_CTX-M-3_	AMK, GEN, KAN, NET, STR, TOB, AMP, PIP, TIC, AMC, CFZ, FEP, CFP, CTX, CXM, CEF, NOR, SUL, TMP, SXT, TET, ATM
EC36	Throat swab	*intI1*	1.9	*dfrA12*-*orfF*-*aadA2*	*bla*_CTX-M-3_	AMK, GEN, KAN, NET, STR, TOB, AMP, PIP, TIC, AMC, CFZ, FEP, CFP, CTX, CXM, CEF, SUL, TMP, SXT, TET
EC37	Throat swab	*intI1*	1.9	*dfrA12*-*orfF*-*aadA2*	*bla*_CTX-M-3_	AMK, GEN, KAN, NET, STR, TOB, AMP, PIP, TIC, CFZ, FEP, CFP, CTX, CXM, CEF, SUL, TMP, SXT, TET, ATM
EC38	Throat swab	–	–	–	–	STR, TET
2008–2010 (31 isolates)						
EC39	Vaginal swab	*intI2*	2.2	*dfrA1*-*sat1*-*aadA1*	*bla*_CTX-M-1_, *bla*_CMY-15_	KAN, STR, AMP, AMC, CFZ, FEP, CFP, CTX, FOX, CAZ, CXM, CEF, CIP, NOR, SUL, TMP, SXT, TET, ATM, NIT
EC40	Tracheostomy tube	–	–	–	–	STR, AMP, CIP, SUL
EC41	Wound	*intI1*	1.9	*dfrA12*-*orfF*-*aadA2*	*bla*_CTX-M-3_, *bla*_TEM-1_	AMK, GEN, KAN, NET, STR, TOB, AMP, PIP, TZP, TIC, AMC, CFZ, FEP, CFP, CTX, FOX,, CXM, CEF, SUL, TMP, SXT, TET, ATM
EC42	Urine	*intI1*	0.55	*dfr2d*	*bla*_CTX-M-3_, *bla*_TEM-1_, *bla*_DHA_	AMK, GEN, KAN, NET, STR, TOB, AMP, PIP, TZP, TIC, AMC, CFZ, FEP, CFP, CTX, FOX, CXM, CEF, CIP, NOR, SUL, TMP, SXT, TET, ATM, CHL
EC43	Throat swab	*intI1*	1.9	*dfrA12*-*orfF*-*aadA2*	*bla*_CTX-M-3_	AMK, GEN, KAN, NET, STR, TOB, AMP, PIP, TIC, AMC, CFZ, FEP, CFP, CTX, CXM, CEF, SUL, TMP, SXT, TET, ATM
EC44	Broncho-alveolar lavage	*intI1*	n.d.	n.d.	*bla*_TEM-1_	AMK, GEN, KAN, NET, STR, TOB, AMP, PIP, TZP, TIC, AMC, CFZ, FEP, CFP, CTX, CAZ, CXM, CEF, SUL, SXT, TET, ATM
EC45	Broncho-alveolar lavage	*intI1*	1.6	*dfrA1*-*aadA1*	*bla*_SHV-12_, *bla*_TEM-1_	AMK, GEN, KAN, NET, STR, TOB, AMP, PIP, TIC, AMC, CFZ, FEP, CFP, CTX, CAZ, CXM, CEF, CIP, NOR, SUL, TMP, SXT, TET, ATM, NIT
EC46	Throat swab	*intI1*	1.9	*dfrA12*-*orfF*-*aadA2*	*bla*_SHV-12_	AMK, GEN, KAN, NET, STR, TOB, AMP, PIP, TIC, AMC, CFZ, FEP, CFP, CTX, CAZ, CXM, CEF, SUL, TMP, SXT, TET, ATM, CHL, NIT
EC47	Urine	*intI1*	n.d.	n.d.	*bla*_TEM-1_	STR, AMP, PIP, TIC, AMC, CFZ, FEP, CFP, CTX, CXM, CEF, CIP, NOR, SUL, TMP, SXT, TET, ATM, CHL
EC48	Vaginal swab	*intI1*	1.7	*dfrA17*-*aadA*5	*bla*_CTX-M-15_, *bla*_TEM-1_, *bla*_OXA-1_	STR, AMP, PIP, TIC, AMC, CFZ, FEP, CFP, CTX, CAZ, CXM, CEF, CIP, NOR, SUL, TMP, SXT, TET, CHL, NIT
EC49	Vaginal swab	*intI1*	1.6	*dfrA1*-*aadA1*	*bla*_CTX-M-15_, *bla*_TEM_	STR, AMP, PIP, TZP, TIC, AMC, CFZ, FEP, CFP, CTX, CXM, CEF, CIP, NOR, SUL, TMP, SXT, TET, ATM
EC50	Ulceration	*intI1*	1.7	*dfrA17*-*aadA*5	*bla*_CTX-M-15_	GEN, KAN, STR, TOB, AMP, PIP, TIC, AMC, CFZ, FEP, CFP, CTX, FOX, CAZ, CXM, CEF, CIP, NOR, SUL, TMP, SXT, TET, ATM, CHL
EC51	Urine	*intI1*	1.0 + 1.7	*aadA1* + *dfrA17*-*aadA*5	*bla*_CTX-M-15_, *bla*_OXA-1_	AMK, GEN, KAN, NET, STR, TOB, AMP, PIP, TZP, TIC, AMC, CFZ, FEP, CFP, CTX, FOX, CAZ, CXM, CEF, CIP, NOR, SUL, TMP, SXT, TET, ATM, CHL, NIT
EC52	Vaginal swab	–	–	–	*bla*_TEM-1_	AMK, KAN, STR, AMP, PIP, TIC, AMC, CFZ, FEP, CFP, CTX, FOX, CXM, CEF, CIP, NOR, SUL, ATM
EC53	Bbroncho-alveolar lavage	*intI1*	1.6	*dfrA1*-*aadA1*	*bla*_CTX-M-15_, *bla*_SHV-12_, *bla*_TEM-1_	AMK, GEN, KAN, NET, STR, TOB, AMP, PIP, TIC, AMC, CFZ, FEP, CFP, FOX, CAZ, CXM, CEF, CIP, NOR, SUL, TMP, SXT, TET, ATM, NIT
EC54	Urine	*intI1*	1.7	*dfrA17*-*aadA*5	*bla*_TEM-1_	GEN, KAN, STR, TOB, AMP, PIP, TIC, AMC, CFZ, FEP, CFP, CTX, FOX, CXM, CEF, CIP, NOR, SUL, TMP, SXT, TET, ATM, CHL
EC55	Urine	–	–	–	–	STR, AMP, PIP, TIC, AMC, CFZ, FEP, CFP, CTX, FOX, CXM, CEF, NOR, SUL, TMP, SXT, TET, ATM, CHL
EC56	Broncho-alveolar lavage	*intI1*	3.0 + 1.6	*aacA4*-*aacC1*-*orfX*-*orfY*-*aadA1* + *dfrA1*-*aadA1*	*bla*_CTX-M-3_, *bla*_SHV-12_, *bla*_TEM-1_	AMK, GEN, KAN, NET, STR, TOB, AMP, PIP, TIC, AMC, CFZ, FEP, CFP, CTX, FOX, CAZ, CXM, CEF, CIP, NOR, SUL, TMP, SXT, TET, ATM, CHL, NIT
EC57	Urine	–	–	–	–	GEN, KAN, STR, AMP, PIP, TIC, AMC, CFZ, FEP, CFP, CTX, FOX, CXM, CEF, SUL, ATM
EC58	Vaginal swab	–	–	–	*bla*_TEM-1_	AMP, PIP, TZP, TIC, AMC, CFZ, FEP, CFP, CTX, FOX, CXM, CEF, SUL, ATM
EC59	Urine	*intI1* + *intI2*	3.0 + 2.2	*aacA4*-*aacC1*-*orfX*-*orfY*-*aadA1* + *dfrA1*-*sat1*-*aadA1* ^a^	*bla*_CTX-M-3_, *bla*_CMY-15_	AMK, GEN, KAN, NET, STR, TOB, AMP, PIP, TZP, TIC, AMC, CFZ, FEP, CFP, CTX, FOX, CAZ, CXM, CEF, CIP, NOR, SUL, TMP, SXT, TET, ATM, CHL, NIT
EC60	Urine	*intI1*	n.d.	n.d.	*bla*_CTX-M-3_	AMK, GEN, KAN, NET, STR, TOB, AMP, PIP, TIC, AMC, CFZ, FEP, CFP, CTX, FOX, CXM, CEF, NOR, SUL, TMP, SXT, TET, ATM
EC61	Urine	*intI1*	1.6	*dfrA1*-*aadA1*	*bla*_CTX-M-55_, *bla*_OXA-1_, *bla*_CMY-15_	AMK, KAN, NET, STR, TOB, AMP, PIP, TIC, AMC, CFZ, FEP, CFP, CTX, FOX, CXM, CEF, CIP, NOR, SUL, TMP, SXT, TET, CHL
EC62	Urine	*intI1*	1.7	*dfrA17*-*aadA*5	*bla*_CTX-M-15_	AMK, GEN, STR, AMP, PIP, TIC, AMC, CFZ, FEP, CFP, CTX, FOX, CAZ, CXM, CEF, CIP, NOR, SUL, ATM, NIT
EC63	Urine	–	–	–	*bla*_CTX-M-15_, *bla*_TEM-1_	AMK, STR, TOB, AMP, PIP, TIC, CFZ, FEP, CFP, CTX, FOX, CXM, CEF, CIP, NOR, SUL, TMP, SXT, TET, ATM, CHL
EC64	Vaginal swab	*intI1*	n.d.	n.d.	–	AMK, GEN, KAN, NET, STR, TOB, AMP, PIP, TIC, CFZ, FEP, CFP, CTX, FOX, CAZ, CXM, CEF, CIP, NOR, SUL, TET, ATM
EC65	Urine	*intI1*	1.7	*dfrA17*-*aadA*5	*bla*_CTX-M-15_	KAN, STR, AMP, PIP, TIC, CFZ, FEP, CFP, CTX, FOX, CXM, CEF, CIP, NOR, SUL, TMP, SXT, TET, ATM
EC66	Urine	*intI1*	0.7	hypothetical protein gene	*bla*_CTX-M-1_, *bla*_OXA-1_	AMK, GEN, KAN, NET, STR, TOB, AMP, PIP, TIC, AMC, CFZ, FEP, CFP, CTX, CAZ, CXM, CEF, CIP, NOR, SUL, TMP, SXT, TET, ATM
EC67	Sputum	*intI1*	0.7	hypothetical protein gene	*bla*_CTX-M-1_, *bla*_OXA-1_	AMK, GEN, KAN, NET, STR, TOB, AMP, PIP, TIC, AMC, CFZ, FEP, CFP, CTX, CAZ, CXM, CEF, CIP, NOR, SUL, TMP, SXT, TET, ATM, NIT
EC68	Urine	*intI1*	0.7	hypothetical protein gene	*bla*_CTX-M-1_, *bla*_OXA-1_	AMK, KAN, NET, STR, TOB, AMP, PIP, TIC, AMC, CFZ, FEP, CFP, CTX, CAZ, CXM, CEF, CIP, NOR, SUL, TMP, SXT, TET, ATM, NIT
EC69	Blood	*intI1*	0.7	hypothetical protein gene	*bla*_CTX-M-1_, *bla*_TEM-1_	KAN, NET, STR, TOB, AMP, PIP, TIC, AMC, CFZ, FEP, CFP, CTX, CAZ, CXM, CEF, CIP, NOR, SUL, TMP, TET, ATM

*AMK* Amikacin, *AMC* amoxicillin-clavulanic acid, *AMP* ampicillin, *ATM* aztreonam, *CFZ* cefazolin, *FEP* cefepime, *CFP* cefoperazone, *CTX* cefotaxime, *FOX* cefoxitin, *CAZ* ceftazidime, *CXM* cefuroxime, *CEF* cephalothin, *CHL* chloramphenicol, *CIP* ciprofloxacin, *GEN* gentamicin, *KAN* kanamycin, *NET* netilmicin, *NIT* nitrofurantoin, *NOR* norfloxacin, *PIP* piperacillin, *TZP* piperacillin-tazobactam, *STR* streptomycin, *SUL* sulfamethoxazole, *TET* tetracycline, *TIC* ticarcillin, *TOB* tobramycin, *TMP* trimethoprim, *SXT* trimethoprim-sulfamethoxazole

*n.d.* Not determined

^a^Class 2 integron

### Antimicrobial susceptibility

**Fig. 1:**
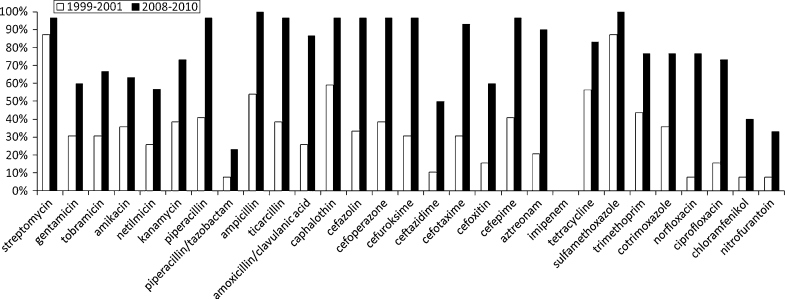
The frequency of antibiotic resistance of *E. coli* strains isolated in 1999–2001 and 2008–2010

Strains isolated in 1999–2001 were resistant towards 1–22 antimicrobials (median 7); the highest frequency of resistance was noted to streptomycin (86.8 %), sulfamethoxazole (86.6 %) cephalotin (57.9 %) and tetracycline (55.3 %), the lowest to imipenem (0 %), norfloxacin (5.3 %), pipercillin/tazobactam and chloramphenicol (7.9 %). The percentage of ESBL-producing isolates was 21.1 %. The frequency of resistance of strains isolated in the beginning and in the end of the decade is shown in Fig. 1.

**Fig. 2:**
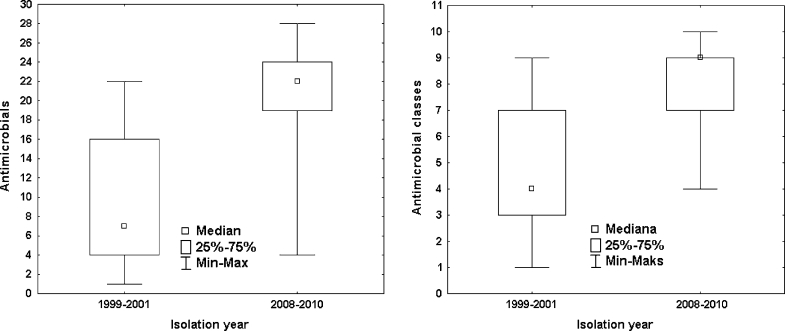
*Box plots* representing antimicrobial resistance ranges of *E. coli* strains isolated in 1999–2001 and 2008–2010

Strains isolated in 2008–2010 had significantly broader resistance ranges defined as the number of antimicrobials to which isolates were resistant and the number of antimicrobial classes to which isolates were resistant (Mann–Whitney *U* test, *P* < 0.001) (Fig. 2). They were resistant towards 4–28 antimicrobials (median 22). The highest resistance percentage was noted for sulfamethoxazole and ampicillin (100 %), streptomycin, cephalothin, cefoperazone, cefuroxime, cefazolin, cephalothin and cefepim (96.8 %). The lowest levels of resistance were recorded for imipenem (0 %), pipercillin/tazobactam (22.6 %) and nitrofurantoin (35.5 %). The percentage of ESBL producers reached 93.5 %.

The frequency of resistance to each of the antimicrobials beside imipenem, streptomycin, piperacillin/tazobactam, and sulfamethoxazole was significantly higher in the second group of isolates (*P* < 0.05).

### Analysis of integrons

We detected integrase genes in 53.6 % isolates (37 strains); 35 had *intI1* gene, one *intI2* gene and one strain had both *intI1* and *intI2* genes (Table 1). We found class 1 integrons in the genomes of 31.6 % strains isolated in 1999–2001, compared to 80.7 % strains isolated in 2008–2010. The frequency of *intI*-positive strains was significantly lower in the first group of isolates (*P* < 0.001). We amplified variable regions of class 1 integrons and detected amplicons of the following sizes: 0.55 kbp (1 strain), 0.7 kbp (4), 1.6 kbp (7), 1.7 kbp (6), 1.9 kbp (11), and 3.0 kbp (1). Two strains had two integrons: 1.0 and 1.7 kbp, and 1.6 and 3.0 kbp (Table 1). We did not manage to receive a CS-PCR product for four *intI1*-positive strains. The two class 2 integrons had variable regions of 2.2 kbp.

The sequence analysis of integrons’ variable regions showed the presence of 1–5 genes. The most often identified gene cassettes were: aminoglycoside adenylyltransferase *aadA* (*aadA1*, *2*, *5*) conferring resistance to aminoglycosides: streptomycin and spectinomycin, aminoglycoside acetyltransferases, *aacA4* and *aacC1,* responsible for resistance to tobramycin, amikacin and gentamicin, respectively, dihydrofolate reductase *dfrA* (*dfrA1*, *12*, *17*, *dfr2d*) conferring resistance to trimethoprim, and streptothricin acetyltransferase *sat2* conferring resistance to streptothricin. We detected the following gene cassette arrays within class 1 integrons: *dfr2d* (0.55 kbp), *aadA1* (1.0 kbp), both present as a single cassette within integron, *dfrA1*-*aadA1* (1.6 kbp), *dfrA17*-*aadA*5 (1.7 kbp), *dfrA12*-*orfF*-*aadA2* (1.9 kbp), and *aacA4*-*aacC1*-*orfX*-*orfY*-*aadA1* (3.0 kbp). Class 2 integron present in the genomes of two strains had *dfrA1*-*sat2*-*aadA1* arrays. In four strains we detected amplicons of 0.7 kbp which were in 99 % identical to a gene coding for hypothetical protein in multiresistant uropathogenic *E. coli* isolated in India (Acc. No CP002797.2) (Table 1).

### Presence of *bla* genes

The strains were examined for presence of genes coding for β-lactamases. Altogether, we identified the following *bla* genes: *bla*_CTX-M-1_-like gene (28 strains), *bla*_TEM_ (20), *bla*_OXA_ (7) and *bla*_SHV_ (5). Sequencing of the amplicons and comparing against GenBank databases identified *bla* genes as *bla*_CTX-M-1_, *bla*_CTX-M-3_, *bla*_CTX-M-15_, *bla*_CTX-M-55_, *bla*_TEM-1_, *bla*_TEM-2_, *bla*_OXA-1_, *bla*_SHV-1_, and *bla*_SHV-12_. Strains harbouring *bla*_CTX-M_ had broader resistance ranges than those without *bla*_CTX-M_ (*P* < 0.001). The distribution of *bla* genes is shown in Table 1.

Eight strains (21.0 %) isolated in 1999–2001 harboured *bla*_CTX-M-3_. Besides, six strains had *bla*_TEM-1_ gene and one isolate *bla*_SHV-1_. In the second group, *bla* genes were found in 29 isolates (96.7 %) and identified as *bla*_CTX-M-3_ (3 isolates), *bla*_CTX-M-15_ (2), *bla*_CTX-M-1_ (1), *bla*_CTX-M-55_ (1), *bla*_TEM-1_ (5), *bla*_OXA-1_ (1) and *bla*_SHV-12_ (1) as single *bla* genes. Eleven ESBL-producing isolates had two *bla* genes whereas three isolates had three *bla* genes (Table 1). In 24 strains (86 %), the genes coding for CTX-M β-lactamase were associated with IS*Ecp1* which was found upstream a *bla*_CTX-M_ gene. The presence of ESBL-producing strains was associated with the time of isolation; there were significantly less ESBL producers among isolates from 1999 to 2001 (*P* < 0.001) and they had significantly lower number of *bla*_CTX-M_ (*P* < 0.001), *bla*_TEM_ (*P* = 0.015) and *bla*_OXA_ (*P* = 0.002) genes. The presence of ESBL phenotype, and ESBL encoding genes (*bla*_CTX-M_ and *bla*_SHV_) was associated with the presence of integrons (*P* < 0.001).

We found AmpC cephalosporinase genes in five strains: four of them had *bla*_CMY_ and one *bla*_DHA_. Sequencing of *bla*_CMY_ and comparing the sequences versus available GenBank data identified them as *bla*_CMY-15_. The *bla*_CMY_ and *bla*_DHA_ genes were present in isolates that had *bla*_CTX-M_ and integrons.

## Discussion

Sixty-nine clinically relevant *E. coli* strains originating from extra-intestinal infections were comprised in the study. We analyzed the level of antimicrobial resistance, the presence of integrons and β-lactamases-coding genes. Comparison of two groups of strains: one isolated in 1999–2001 and another in 2008–2010 showed significant differences in drug resistance frequency, presence of integrons and β-lactamase-coding genes. The frequency of antimicrobial resistance to all antimicrobials beside imipenem, streptomycin, piperacillin/tazobactam, and sulfamethoxazole increased significantly, reaching high levels toward aminoglycosides, β-lactams and fluoroquinolones. It generally mirrors the trends in *E. coli* resistance, yet we must emphasize the fact that the frequency of resistance of strains isolated in 1999–2001 was already relatively high compared to other data. Turner (2008) has reported results of Meropenem Yearly Susceptibility Test Information Collection (MYSTIC) program from 2006 and its comparison with 2002. All antibiotics demonstrated reduction in activity against clinical isolates of *E. coli* in 2006 in comparison with the results from 2002: for gentamicin the percentage of sensitive strains was 91.7 % (86.7 % in 2002), tobramycin 69.6 % (84.2 %) ciprofloxacin 73.3 % (82.1 %), ceftazidime 86.6 % (92.5 %), piperacillin + tazobactam 85.9 % (93.1 %), imipenem 99.7% (99.4 %) and amikacin 99 % (not tested in 2002). According to data collected by the European Antimicrobial Resistance Surveillance System (EARSS and EARS-Net) regarding *E. coli* isolated from bloodstream infections, a significant increase of resistance in years 2002–2009 has been observed (Gagliotti et al. 2011). That regarded resistance to third-generation cephalosporins and combined resistance, i.e. resistance to two, three or four antimicrobials classes (aminoglycosides, aminopenicilins, third-generation cephalosporins and fluoroquinolones). We noted an increase in resistance to aminoglycosides, beta-lactams and fluoroquinolones. The EARSS survey from 2008 has reported the resistance against third-generation cephalosporines to be the most dynamic in Europe, which predicted increase in the number of ESBL-producing strains (European Centre for Disease Prevention and Control 2009). The EARS-Net report from 2010 has supported the remarkable Europe-wide decline of antimicrobial susceptibility in *E. coli*: in several countries both multidrug resistance and resistance frequency were increasing. The proportion of *E. coli* isolates resistant to third-generation cephalosporins increased significantly during 2006–2010 in half of the reporting countries. Among these isolates, a high proportion (65–100 %) was identified as ESBL producers. These data indicate that ESBL production is highly prevalent in third-generation cephalosporin-resistant *E. coli* in European hospitals (European Centre for Disease Prevention and Control 2011). In our research, the percentage of ESBL-positive strains increased significantly from 21.1 to 93.5 % between 1999–2001 and 2008–2010. Most of the ESBL-producing isolates (75.7 %) had a *bla*_CTX-M_ gene: the frequency of strains with *bla*_CTX-M_ grew significantly in group 2 in comparison with group 1. The analysis of the resistance frequency and the presence of *bla*_CTX-M_ genes reflected another tendency: the strains producing CTX-M β-lactamases acquired resistance to other than β-lactams, classes of antimicrobials namely tetracycline (*P* = 0.002) and fluoroquinolones (*P* < 0.001). Surveys conducted worldwide have shown a growing resistance frequency to antimicrobials like tetracycline, gentamicin, tobramycin and ciprofloxacin in CTX-M producing *E. coli* (Pitout and Laupland 2008).

We also noticed the increase in the number of strains with integrons from 31.6 to 80.7 %. The presence of integrase genes was associated with increased frequency of resistance to each antimicrobial tested besides imipenem, piperacillin/tazobactam and ceftazidime (*P* < 0.05). The presence of integrons was also associated with multidrug resistance and the presence of ESBL phenotype and ESBL-encoding genes (*P* < 0.001). The genetic content of integrons comprised genes determining resistance toward aminoglycosides, sulfonamides and trimethoprim but the resistance of *intI*-positive isolates was far broader. In the first group of *E. coli* we detected three arrays of genes within integrons: *dfrA1*-*aadA, dfrA17*-*aadA*5, and *dfrA12*-*orfF*-*aadA2*. Gene cassette arrays like that are commonly appearing in *E. coli* isolates (Machado et al. 2005; El-Najjar et al. 2010). In the genomes of isolates from 2008 to 2010 we observed greater versatility of gene cassettes, more gene cassettes within an integron, appearance of class 2 integrons, and presence of more than one integron in bacterial genome. We detected six different integron-embedded gene cassette arrays: *dfr2d*, *aadA1*, *dfrA1*-*aadA1*, *dfrA17*-*aadA*5, *dfrA12*-*orfF*-*aadA2*, and *aacA4*-*aacC1*-*orfA*-*orfB*-*aadA1* as well as cassettes coding for hypothetical proteins. All *intI1*-positive strains produced ESBL. The most often identified gene determining β-lactamase production was *bla*_CTX-M_ type, identified by sequencing as *bla*_CTX-M-1_, *bla*_CTX-M-3_, *bla*_CTX-M-15_, and *bla*_CTX-M-55_. In strains isolated from 1998 to 2001, only *bla*_CTX-M-3_ was present. The CTX-M β-lactamases are now predominant in Poland and were noted for the first time in the late 1990s and identified as CTX-M-3. (Gniadkowski et al. 1998b; Livermore et al. 2007). In the following years, *bla*_CTX-M-15_ appeared possibly by point mutation in *bla*_CTX-M-3_ (Poirel et al. 2002). The CTX-M-15 lactamase is 100-fold more active against ceftazidime than CTX-M-3 (Cartelle et al. 2004).

The genetic environment of bla_CTX-M_ genes in most of our isolates was homogenous and consisted of *ISEcp1* upstream *bla*_CTX-M-1_, *bla*_CTX-M-3_, and *bla*_CTX-M-15_. *ISEcp1* is frequently associated with *bla*_CTX-M_ genes and as it includes promoter sequences, it enhances otherwise poor *bla*_CTX-M_ expression (Poirel et al. 2008). That element may also transpose downstream located fragments and thus facilitate the spread of *bla*_CTX-M_ genes (Partridge 2011). In the isolates from 2008 to 2010 beside integrons and *bla*_CTX-M_, there were also plasmid-mediated AmpC β-lactamases: *bla*_CMY-15_ and *bla*_DHA_. AmpC β-lactamases at high levels, hydrolyse penicillins, most cephalosporins, cephamycins and monobactams (Pitout 2012). In a survey comprising 13 Polish hospitals, *bla*_CMY_ were identified in *Proteus mirabilis* only, and CMY-15 was the type of enzyme most common among them (Empel et al. 2008).

In summary, we found significant increase in resistance frequency including resistance to first line antibiotics like cephalosporins and fluoroquinolones, integron presence, and ESBL phenotype frequency. We also noticed significant increase in the frequency of *bla*_CTX-M_ β-lactamases with appearance of *bla*_CTX-M-15_ variant and newer plasmid-encoded β-lactamases like CMY and DHA. We observed the emergence of strains with resistance to several classes of antimicrobials simultaneously with integrons, ESBL and AmpC β-lactamases coding genes. That may predict the spread of strains resistant to main classes of antimicrobials with no options for treatment apart from monobactams.

## Electronic supplementary material

Below is the link to the electronic supplementary material.

Supplementary material 1 (TIFF 3901 kb)
