# Supplementary figures and images for: Increased frequency of integrons and β-lactamase-coding genes among extraintestinal Escherichia coli isolated with a 7-year interval
Source: Antonie Van Leeuwenhoek. 2012 Sep 4;103(1):163–74. doi: 10.1007/s10482-012-9797-9 (PMC3528966; doi:10.1007/s10482-012-9797-9)

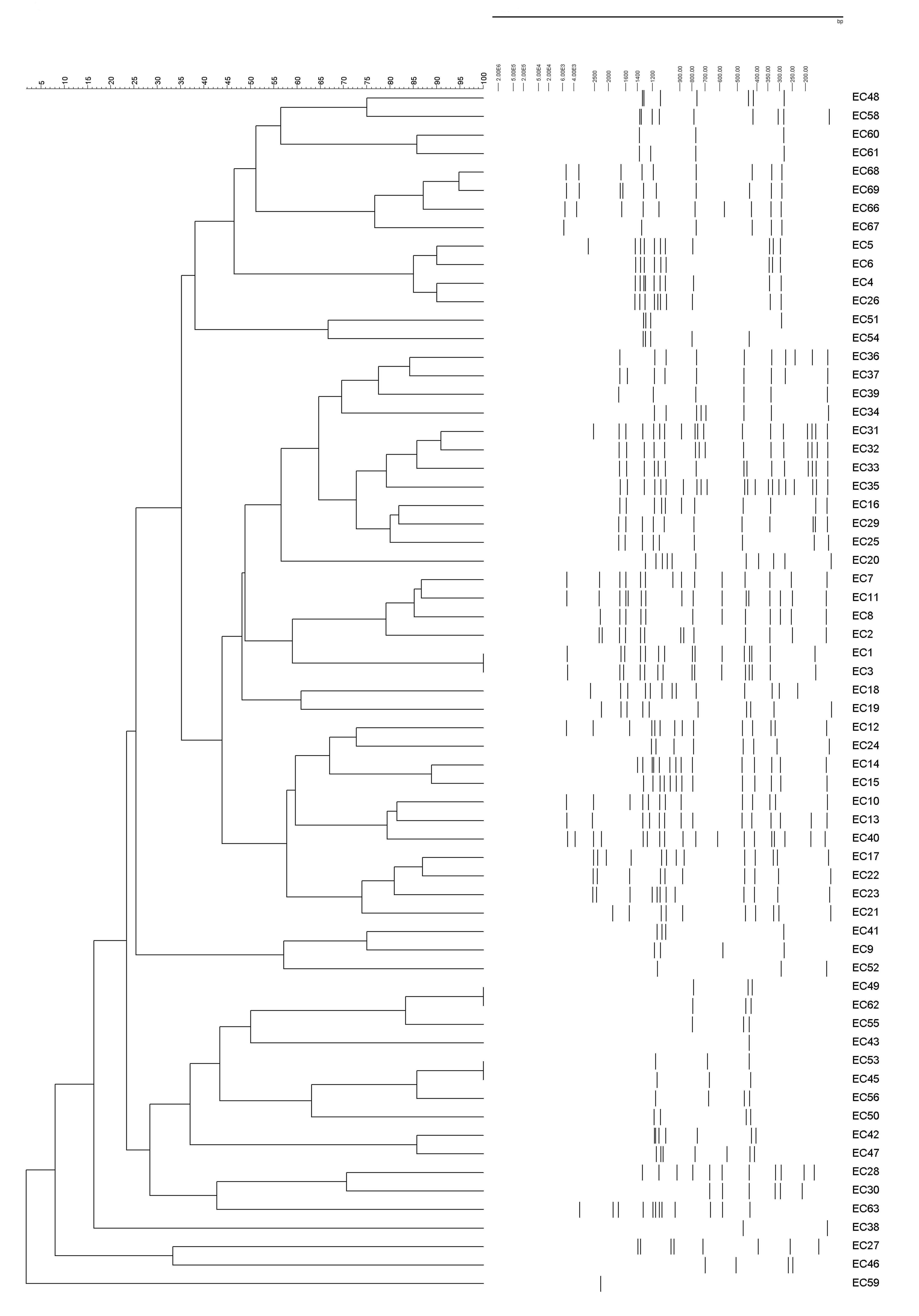

Supplement: Supplementary file 1 — Supplementary material 1 (TIFF 3901 kb) [file 10482_2012_9797_MOESM1_ESM.tif]
